# Long-term pulmonary outcomes of young adults born prematurely: a Polish prospective cohort study PREMATURITAS 20

**DOI:** 10.1186/s12890-024-02939-5

**Published:** 2024-03-12

**Authors:** Katarzyna Walicka-Serzysko, Magdalena Postek, Urszula Borawska-Kowalczyk, Katarzyna Szamotulska, Piotr Kwaśniewicz, Krystyna Polak, Ewa Mierzejewska, Dorota Sands, Magdalena Rutkowska

**Affiliations:** 1 Cystic Fibrosis Department, Institute of Mother and Child, Warsaw, Poland; 2 Cystic Fibrosis Centre, Pediatric Hospital, Dziekanów Leśny, Poland; 3 Department of Epidemiology and Biostatistics, Institute of Mother and Child, Warsaw, Poland; 4 Diagnostic Imaging Department, Institute of Mother and Child, Warsaw, Poland; 5 Neonatology Clinic, Institute of Mother and Child, Warsaw, Poland

**Keywords:** Preterm young adults, Pulmonary function test, Lung clearance index, Health related quality of life, Anxiety, Depression

## Abstract

**Background:**

The long-term consequences of prematurity are often not sufficiently recognized. To address this gap, a prospective cohort study, which is a continuation of the multicenter Polish study PREMATURITAS, was conducted, utilizing unique clinical data from 20 years ago.

**Objective:**

The main goal was to evaluate lung function, detect any structural abnormalities using lung ultrasound, and assess psychological well-being in young adults born between 24 and 34 weeks of gestational age (GA). Additionally, the study aimed to investigate potential associations between perinatal risk factors and abnormalities observed in pulmonary function tests (PFTs) during adulthood.

**Methods:**

The young survivors underwent a comprehensive set of PFTs, a lung ultrasound, along with the quality of life assessment. Information regarding the neonatal period and respiratory complications was obtained from the baseline data collected in the PREMATURITAS study.

**Results:**

A total of 52 young adults, with a mean age of 21.6 years, underwent PFTs. They were divided into two groups based on GA: 24–28 weeks (*n* = 12) and 29–34 weeks (*n* = 40). The subgroup born more prematurely had significantly higher lung clearance index (LCI), compared to the other subgroup (*p* = 0.013). LCI ≥ 6.99 was more frequently observed in the more premature group (50% vs. 12.5%, *p* = 0.005), those who did not receive prenatal steroids (*p* = 0.020), with a diagnosis of Respiratory Distress Syndrome (*p* = 0.034), those who received surfactant (*p* = 0.026), and mechanically ventilated ≥ 7 days (*p* = 0.005). Additionally, elevated LCI was associated with the diagnosis of asthma (*p* = 0.010).

**Conclusions:**

The findings suggest pulmonary effects due to prematurity persist into adulthood and their insult on small airway function. Regular follow-up evaluations of young survivors born preterm should include assessments of PFTs. Specifically, the use of LCI can provide valuable insights into long-term pulmonary impairment.

## Background

While advances in neonatal care have resulted in improved survival rates of premature infants, the impacts on long-term outcomes are poorly defined. Despite several lung-protective strategies, the incidence of bronchopulmonary dysplasia (BPD) has remained the same [1, 2] or increased [3]. Additionally, late outcomes remain challenging as rapid advances in medical management result in current young adult preterm survivors representing outdated neonatal care. While pulmonary symptoms decrease with growth among very preterm babies, long-term follow-up studies raise concerns for persistent pulmonary dysfunction along with asthma-like symptoms in comparison with term controls [4–6]. In addition to the spirometric parameters, lung clearance index (LCI), a marker of ventilation inhomogeneity derived from multiple breath washout (MBW), start to be applied in preterm subjects [7]. In certain studies, LCI was found to be higher in children born very preterm [8]. Nevertheless, further research is required, particularly among young survivors.

The group of extremely preterm newborns and very preterm, whose survival rate increased mainly at the end of the 20th century, became a group requiring special observation and long-term development assessment. This is mainly due to the results of both the American, Australian, and the two largest European studies (EPIPAGE1-France, EPICURE1-England), which showed the presence of significant disorders of respiratory, motor, cognitive, emotional, and social development at the age of 6 to 19 in this population of children [9–12].

In Poland, a similar multicentre cohort study called PREMATURITAS was conducted by the Institute of Mother and Child. It covered all newborns born at gestational age (GA) 24–32 weeks in the years 1998–1999 in Warsaw (*n* = 307). Overall, 186 children were discharged from hospital after birth. The development of 162 at 2 years of age and 126 at 6 years of age has been previously described [13, 14]. The current study is a continuation of PREMATURITAS.

The main objective of this study was to assess lung function in very preterm patients during young adulthood, using the latest pulmonary function tests (PFTs) and lung ultrasound. Additionally we wanted to evaluate whether the respiratory functioning of young survivors was related with their subjective assessment of quality of life (QoL) and the presence of symptoms of depression and anxiety.

One of the detailed aims was to investigate the relationship between perinatal risk factors and the observed changes in PFTs. We hypothesised that adults born preterm would exhibit lung function impairment, particularly in terms of airway obstruction and ventilation inhomogeneity. Furthermore, we expected that LCI would serve as a sensitive marker for detecting lung function abnormalities in adult survivors.

## Methods

### Study design

The Polish study PREMATURITAS 20 was carried out at the Institute of Mother and Child (grant No 510-04-10) from July 2020 to September 2021. Participants are survivors of the cohort PREMATURITAS contacted at age 21–24 years.

Young adults were enrolled based on the inclusion criteria (members of the PREMATURITAS cohort) and the following exclusion criteria: inability to perform PFTs (spirometry, plethysmography, impulse oscillometry (IOS), multiple breath nitrogen washout (MBNW), and fractional exhaled nitric oxide (FeNO ), lack of cooperation including patients with severe cerebral palsy, current pulmonary infection, clinical condition precluding a patient from performing pulmonary function tests, e.g., dyspnea, hemoptysis, and other severe pulmonary complications.

During the visits, after obtaining informed consent, patients were clinically assessed, including anthropometric and pulmonary function tests (consecutively FeNO, IOS, MBNW, spirometry, plethysmography), lung ultrasound and quality of life and mental health measures. Due to the Covid-19 pandemic, all precautions have been taken during visits. Before the visit, every participant was asked about symptoms of respiratory infection. Based on history and physical examination, the tests were performed in a stable condition to avoid the influence of acute infection (including SARS-CoV-2) on results.

Information about prenatal steroids, GA, birthweight, and neonatal respiratory complications, such as Respiratory Distress Syndrome (RDS), mechanical ventilation, surfactant and postnatal steroids administration, infections, and hospitalisation up to 2 years of age was obtained from the PREMATURITAS study data collected at baseline and follow-up.

The protocol was approved on April 6, 2020, by the local ethics committee at the Institute of Mother and Child in Warsaw (opinion number 5/2020). The participants were enrolled in the study upon providing their informed consent.

### Pulmonary function measurements

Lung function measurements were carried out according to the standard European Respiratory Society (ERS) guidelines for lung function measurements. All types of equipment were calibrated every day before tests. During the calibration spirometer and plethysmographic cabin with a 3 L syringe, the error in measuring the linearity and repeatability did not exceed ± 2.5%. Ambient temperature, barometric pressure, and saturated with water vapor were recorded every day before measurements. Patients performed spirometry in a sitting position. They accomplished at least three reproducible trials. Test results were considered reproducible if the difference between the two largest forced vital capacity (FVC) values was 0.100 L, there was no cough in the first second of expiration, no glottic closure after 1 s of expiration, no evidence of obstruction in the mouthpiece or spirometer, and no evidence of a leak reaching the expiratory plateau within 15 s [15, 16].

IOS tests were performed in a sitting position and met the requirements specified in the American Thoracic Society (ATS) guidelines [17]. The minimum number of technically acceptable trials was 3 and the coefficient of variation between replicates was ⩽10% for adults. We used the Vyntus IOS device (CareFusion, Hochberg, Germany) for impulse oscillometry and spirometry.

The whole-body plethysmography is performed in a lockable airtight cabin. At least 3 repeatable and acceptable maneuvers are carried out. The plethysmography was performed using Master Screen Body/Diff Jaeger (CareFusion, Hochberg, Germany) in pursuance of the ATS/ERS criteria [18]. Spirometry and flow-volume curves were acquired by whole‐body plethysmography. Lung function results were recorded and analysed according to the Global Lung Function Initiative (GLI) [15].

Quantification of ventilation inhomogeneity based on LCI was measured by the MBNW technique. MBNW tests were performed using the Exhalyzer- D (EcoMedics AG, Duernten, Switzerland, software version 3.3.1). According to the ERS/ATS guidelines, the MBNW test was considered successful if there were at least two or more technically acceptable manoeuvers [19]. Trials were defined as a good quality not only by the software but also included close observation by the operator of the subject’s behavior during testing. LCI was expressed as the mean of a minimum of two technically acceptable results obtained during one session. The upper limit of normal for LCI ≥ 6,99 was determined based on normative data for MBNW with correction for sensor error [20, 21]. To avoid affecting the airway caliber by forced expiratory maneuvers MBNW and IOS were performed before plethysmography/spirometry.

Nitric oxide concentration (Medisoft FENO + analyser) at 50 ml/s exhalation flow rates was measured. Calibration of the FENO analyser is performed by professional service twice a year.

### Lung ultrasound

Lungs were assessed with the use of Samsung RS 85 ultrasound both convex (CA1-7 S) and linear (LA4-18B) probes using a modified Copetti method [22] with additional transhepatic and transsplenial scans [23]. Artifact reduction modes were turned off and the mechanical index was set up to be lower than 0.4 due to safety reasons [24].

### Quality of life and mental health measures

The World Health Organization Quality-of-Life Scale (WHOQOL-BREF) is a 26-item generic questionnaire used to assess the quality of life in four domains: physical health, psychological, social relationships and environment [25, 26]. Responses are rated on a 5-point Likert scale, with a maximum score of 20 points for each domain. Higher scores indicate better QoL. In addition, two general questions provide information on global QoL (Q1) rated on a scale from ‘very poor’ to ‘very good’, and health satisfaction (Q2), rated on a scale from ‘very dissatisfied’ to ‘very satisfied’. These questions are analysed separately, with responses divided into two groups: ‘dissatisfied’ for answers from 1 to 3 and ‘satisfied’ for answers from 4 to 5. The time frame for responses is the previous two weeks.

The Polish version of the Patient Health Questionnaire PHQ-9 [27] developed by the MAPI Research Institute and available at www.phqscreeners.com, was used. It is a screening tool used to assess the severity of depression symptoms. It consists of 9 main questions and one supplementary question. The essential questions focus on the frequency of occurrence of depressive symptoms in the past two weeks, as described in the diagnostic criteria of DSM-IV. The answers range on a scale from 0 (‘not at all’) to 3 (‘nearly every day’), with a maximum possible score of 27. In Poland, Kokoszka et al. established an optimal cutoff point of > 12, indicating clinically significant severity of depressive symptoms [28].The additional question, which is not included in the total score, assesses the extent to which the symptoms have interfered with the functioning of the individual.

The Polish version of the Generalized Anxiety Disorders (GAD)-7 [29] available at www.phqscreeners.com, was used. This screening measure contains 7 items and is developed to assess the presence and severity of anxiety symptoms. Participants respond to the questions on a four-point scale, ranging from 0 (‘not at all’) to 3 (‘nearly every day’). Higher results indicate a greater presence of symptoms of generalized anxiety disorder, with a maximum of 21 points. A score > 10 suggests a clinically significant severity of generalized anxiety disorder.

### Statistical analysis

Descriptive and inferential statistics were used in statistical analysis. The results are presented as means ± standard deviations (SD) or proportions. The Kolmogorov-Smirnov test was used for evaluating distributions for normality. Differences between groups were assessed using Student’s t-test for normally distributed data and non-parametric Mann-Whitney test for non-normally distributed parameters. Chi-square test (or Fisher test) was applied to verify hypotheses regarding associations between independent categorical variables. A P value < 0.05 was considered to be statistically significant. Statistical analysis was performed using IBM SPSS v. 25.0 software.

## Results

### Subjects characteristics

According to the project schedule we contacted 66 young adults at the age of 21–24 years (52.4% of the attendees who took part in the study at the age of 6). Over the 1-year recruitment period, 52 young adults performed PFTs tests. Fourteen out of sixty-six contacted patients either did not sign up for the visit or fulfilled any of the exclusion criteria. The mean age of the studied group was 21.6 ± 0.6 years. The female gender slightly prevailed (53.8%). The smallest patient was born at GA at 25 weeks and weighed 560 g. A total of 12 (23.1%) of the respondents were born at GA ≤ 28 weeks, 33 (63.5%) were born between 29 and 32 weeks, and the remaining 7 (13.5%) were born 33–34 weeks. BPD was diagnosed in 4 patients but only one underwent pulmonary function tests. Characteristics of the study group is outlined in Table 1.

**Table 1:** Demographic and perinatal characteristics of the study group

Measure	All subjects (*n* = 52)	Gestational age	
		24–28 weeks (*n* = 12)	29–34 weeks (*n* = 40)
Sex [*n* (%)]			
Male	24 (46.2%)	7 (58.3%)	17 (42.5%)
Female	28 (53.8%)	5 (41.7%)	23 (57.5%)
Age at assessment, years [mean ± SD]	21.6 ± 0.6	21.6 ± 0.8	21.6 ± 0.5
Height, cm [mean ± SD]			
Male	174.2 ± 9.2	171.9 ± 11.8	175.1 ± 8.1
Female	163.5 ± 6.0	163.9 ± 6.5	163.4 ± 6.1
Weight, kg [mean ± SD]			
Male	70.5 ± 15.4	74.3 ± 22.9	69.0 ± 11.7
Female	61.8 ± 16.1	63.2 ± 11.5	61.5 ± 17.1
BMI [mean ± SD]			
Male	23.1 ± 3.6	24.7 ± 4.6	22.4 ± 3.0
Female	23.1 ± 5.8	23.4 ± 3.3	23.0 ± 6.3
Gestational age, weeks [mean ± SD]	30.4 ± 2.3	27.1 ± 1.2	31.4 ± 1.4
Birth weight, g [mean ± SD]	1482.9 ± 394.0	1066.7 ± 242.2	1607.8 ± 342.3
Prenatal steroids [n (%)]	38 (73.1%)	7 (58.3%)	31 (77.5%)
Congenital pneumonia [n (%)]	18 (34.6%)	8 (66.7%)	10 (25.0%)
Mechanical ventilation ≥ 7 days [n (%)]	12 (23.1%)	7 (58.3%)	5 (12.5%)
Surfactant [n (%)]	11 (21.2%)	8 (66.7%)	3 (7.5%)
RDS [n (%)]	15 (28.8%)	9 (75.0%)	6 (15.0%)
Sepsis [n (%)]	6 (11.5%)	4 (33.3%)	2 (5.0%)
Postnatal steroids [n (%)]	4 (7.8%)	3 (25.0%)	1 (2.6%)
Hospitalisations due to respiratory infections up to 2 years of age [n (%)]	12 (23.1%)	3 (25.0%)	9 (22.5%)
Current asthma [n (%)]	6 (11.5%)	1 (8.3%)	5 (12.5%)
Current smoking [n (%)]	15 (28.8%)	3 (25.0%)	12 (30.0%)

Based on their gestational age, the subjects were classified into 2 subgroups: born between 24 and 28 weeks and born between 29 and 34 weeks of gestation. Respiratory complications such as RDS, mechanical ventilation, congenital pneumonia, sepsis, the need for surfactant and postnatal steroids treatment as well as hospitalisation up to 2 years of age were more common in the group of extremely immature children.

### Lung function evaluation in early adulthood

#### Airway obstruction

Airway obstruction defined as forced expiratory volume in the first second (FEV_1_) /FVC≤-1.646 z-score was observed in 28.0% of patients: in 50.0% of subjects born at GA 24–28 weeks and in 21.1% of subjects born at GA 29–34 weeks (Table 2). The difference between GA groups was at the border of statistical significance (*p* = 0.052).

**Table 2:** Pulmonary function tests’ results in prematurely born young adults and gestational age at birth

Measure	Total (*n* = 52)	Gestational age		
		24–28 weeks (*n* = 12)	29–34 weeks (*n* = 40)	p-value
FEV_1_, %	94.3 ± 14.28	93.00 ± 16.03	94.71 ± 13.89	0.721
FEV_1_, z-score	-0.39 ± 1.34	-0.60 ± 1.38	-0.32 ± 1.33	0.547
FEV_1_ < 80%pred.	16.0%	25.0%	13.2%	0.329
FEV_1_/FVC, %	91.18 ± 11.6	86.08 ± 12.29	92.79 ± 11.06	0.081
FEV_1_/FVC, z-score	-0.88 ± 1.39	-1.53 ± 1.24	-0.68 ± 1.38	0.062
FEV_1_/FVC z-score ≤-1.646	28.0%	50.0%	21.1%	0.052
MEF 25, %	88.84 ± 37.73	76.17 ± 31.39	92.74 ± 39.00	0.186
MEF 25, z-score	-0.53 ± 1.23	-0.93 ± 1.15	-0.41 ± 1.24	0.199
MEF 50, %	89.80 ± 29.84	81.75 ± 30.02	92.28 ± 29.73	0.290
MEF 50, z-score	-0.54 ± 1.39	-0.92 ± 1.46	-0.43 ± 1.36	0.284
MEF 75, %	82.25 ± 21.63	76.75 ± 25.85	83.95 ± 20.24	0.318
MEF 75, z-score	-0.83 ± 1.02	-1.10 ± 1.23	-0.75 ± 0.95	0.311
PEF, %	82.78 ± 17.77	78.08 ± 23.22	84.23 ± 15.82	0.299
PEF z-score	-1.37 ± 1.42	-1.73 ± 1.87	-1.26 ± 1.26	0.325
TLC, %	107.40 ± 10.63	106.27 ± 9.50	107.72 ± 11.03	0.695
TLC z-score	0.67 ± 0.95	0.59 ± 0.80	0.69 ± 1.00	0.755
RV, %	122.22 ± 27.03	116.75 ± 23.74	123.90 ± 28.04	0.429
RV z-score	0.86 ± 1.07	0.63 ± 0.88	0.93 ± 1.12	0.403
RV/TLC, %	112.98 ± 26.17	109.18 ± 28.18	114.05 ± 25.86	0.591
RV/TLC z-score	0.58 ± 1.17	0.40 ± 1.23	0.64 ± 1.16	0.558
FRC, %	109.76 ± 17.57	115.83 ± 16.10	107.90 ± 17.78	0.174
FRC z-score	0.51 ± 0.95	0.76 ± 0.79	0.44 ± 0.99	0.313
LCI 2.5%norm	6.52 ± 0.62	6.90 ± 0.67	6.40 ± 0.56	0.013
LCI 2.5%norm ≥6.99	21.2%	50.0%	12.5%	0.005
R5Hz	0.35 ± 0.09	0.36 ± 0.08	0.35 ± 0.09	0.965
R20Hz	0.32 ± 0.07	0.31 ± 0.07	0.32 ± 0.07	0.474
R5-R20	0.04 ± 0.04	0.05 ± 0.05	0.03 ± 0.04	0.338
X5	-0.10 ± 0.05	-0.09 ± 0.08	-0.10 ± 0.03	0.519
AX	0.33 ± 0.30	0.36 ± 0.21	0.33 ± 0.33	0.236
Fres (IOS)	11.86 ± 4.22	12.76 ± 3.40	11.59 ± 4.44	0.405
FeNO	19.34 ± 14.93	18.45 ± 11.9	19.59 ± 15.80	0.826
FeNO > 25 ppb	24.0%	27.3%	23.1%	0.774

### Ventilation inhomogeneity (VI)

Ventilation inhomogeneity described as LCI ≥ 6,99 was presented in 21.2% of adults: in 50.0% of those born at GA 24–28 weeks and 12.5% of those born at GA 29–34 weeks (Table 2). LCI was on average significantly higher in the more preterm group compared to the other (*p* = 0.013). LCI ≥ 6,99 was reported in 14.3% of subjects with FEV_1_ ≥ 80%pred and in 50% with FEV_1_ < 80%pred.(*p* = 0.021). Subjects with LCI ≥ 6.99 had significantly higher residual volume to total lung capacity ratio (RV/TLC) percent and z-score: 131.11 ± 37.11 vs. 109.00 ± 21.75 (*p* = 0.020) and 1.36 ± 1.61 vs. 0.41 ± 0.99 (*p* = 0.026), respectively.

### IOS

We haven’t found a significant association between R5Hz, R20Hz, X5, R5-R20, Fres and AX values and gestational age (Table 2). However, Fres values were higher in the group born extremely premature than in the group more mature The increased value of the Fres was significantly correlated with the decreased FEV1 / FVC z-score (*r*=-0.392, *p* = 0.005).

### Neonatal and infant risk factors of respiratory dysfunction in early adulthood

#### Pre- and postnatal steroids

The provision of prenatal steroid was associated with a lower incidence of pulmonary dysfunction in young adults, as demonstrated by LCI ≥ 6.99 (13.2%), compared to subjects not receiving such treatment (42.9%, *p* = 0.020). There was also observed the trend toward higher values of FEV_1_ z-score (-0.16 vs. -0.97, *p* = 0.053) in individuals receiving steroids before birth (Table 3).

Two of three patients (67%) who received postnatal steroids had FEV_1_ < 80% pred compared to those who did not receive such treatment (13.0%) (*p* = 0.015). Two of these patients also had features of obstruction (FEV_1_ / FVC ≤-1.646 z-score) and more often LCI ≥ 6.99 (50.0%) compared to the group not treated with steroids in the neonatal period: 26.1% and 19.1%, respectively. Subjects receiving postnatal steroids had on average lower FEV_1_/FVC z-score and maximal expiratory flow at 75% of vital capacity (MEF 75)-z-score values than other patients (*p* = 0.042 and *p* = 0.093, respectively, Table 3).

**Table 3:** Perinatal and adulthood risk factors and standardised spirometric and MBNW tests’ results in prematurely born young adults

	FEV1 z -score (*n* = 50)		FEV1/FVC z -score (*n* = 50)		MEF25 z -score (*n* = 51)		MEF50 z -score (*n* = 51)		MEF75 z -score (*n* = 51)		LCi 2.5%norm (*n* = 52)		LCI 2.5%norm. ≥ 6.99		
	mean ± SD	p-value	mean ± SD	p-value	mean ± SD	p-value	mean ± SD	p-value	mean ± SD	p`-value	mean ± SD	p-value	n	%	p-value
Prenatal steroids															
No (*n* = 14)	-0.97 ± 1.14	0.053	-0.86 ± 1.61	0.947	-0.71 ± 1.13	0.522	-0.74 ± 1.27	0.533	-0.98 ± 0.95	0.530	6.78 ± 0.64	0.058	14	42.9%	0.020
Yes (*n* = 38)	-0.16 ± 1.35		-0.89 ± 1.32		-0.46 ± 1.28		-0.47 ± 1.44		-0.78 ± 1.05		6.42 ± 0.59		38	13.2%	
Congenital pneumonia															
No (*n* = 34)	-0.32 ± 1.41	0.593	-0.85 ± 1.51	0.803	-0.62 ± 1.36	0.500	-0.62 ± 1.53	0.607	-0.80 ± 1.09	0.730	6.47 ± 0.56	0.501	34	14.7%	0.159
Yes (*n* = 18)	-0.53 ± 1.21		-0.95 ± 1.17		-0.37 ±		-0.41 ± 1.12		-0.90 ± 0.89		6.60 ± 0.72		18	33.3%	
Mechanical ventilation															
< 7 days (*n* = 40)	-0.39 ± 1.38	0.968	-0.82 ± 1.42	0.503	-0.60 ± 1.27	0.433	-0.59 ± 1.46	0.680	-0.74 ± 1.07	0.240	6.39 ± 0.53	0.005	40	12.5%	0.005
≥ 7 days (*n* = 12)	-0.37 ± 1.20		-1.15 ± 1.29		-0.27 ± 1.11		-0.33 ± 1.11		-1.15 ± 0.76		6.94 ± 0.72		12	50.0%	
Surfactant															
No (*n* = 41)	-0.37 ± 1.32	0.834	-0.79 ± 1.33	0.373	-0.53 ± 1.23	0.947	-0.54 ± 1.35	0.943	-0.75 ± 0.93	0.342	6.40 ± 0.52	0.010	41	14.6%	0.026
Yes (*n* = 11)	-0.47 ± 1.48		-1.24 ± 1.63		-0.56 ± 1.33		-0.57 ± 1.61		-1.11 ± 1.35		6.94 ± 0.78		11	45.5%	
RDS															
No (*n* = 37)	-0.44 ± 1.36	0.685	-0.75 ± 1.36	0.298	-0.57 ± 1.23	0.729	-0.52 ± 1.40	0.860	-0.73 ± 0.97	0.253	6.42 ± 0.53	0.081	37	13.5%	0.034
Yes (*n* = 15)	-0.27 ± 1.32		-1.21 ± 1.45		-0.43 ± 1.28		-0.60 ± 1.41		-1.10 ± 1.13		6.75 ± 0.77		15	40.0%	
Sepsis															
No (*n* = 46)	-0.42 ± 1.32	0.677	-0.87 ± 1.43	0.874	-0.54 ± 1.27	0.951	-0.58 ± 1.40	0.621	-0.86 ± 0.99	0.613	6.46 ± 0.59	0.085	46	19.6%	0.595
Yes (*n* = 6)	-0.17 ± 1.54		-0.96 ± 1.18		-0.50 ± 0.96		-0.28 ± 1.35		-0.63 ± 1.33		6.93 ± 0.72		6	33.3%	
Postnatal steroids															
No (*n* = 47)	-0.30 ± 1.34	0.107	-0.76 ± 1.36	0.042	-0.49 ± 1.21	0.617	-0.47 ± 1.37	0.294	-0.76 ± 1.00	0.093	6.49 ± 0.61	0.104	47	19.1%	0.150
Yes (*n* = 4)	-1.60 ± 1.02		-2.45 ± 1.17		-0.82 ± 1.76		-1.25 ± 1.72		-1.66 ± 1.19		7.01 ± 0.59		4	50.0%	
Hospitalisations due to respiratory infections up to 2 years of age															
No (*n* = 40)	-0.22 ± 1.10	0.103	-0.61 ± 1.30	0.013	-0.29 ± 1.13	0.010	-0.25 ± 1.24	0.006	-0.62 ± 0.92	0.006	6.47 ± 0.59	0.294	40	22.5%	0.664
Yes (*n* = 12)	-0.94 ± 1.85		-1.74 ± 1.35		-1.32 ± 1.27		-1.48 ± 1.47		-1.52 ± 1.06		6.68 ± 0.72		12	16.7%	
Current asthma															
No (*n* = 46)	-0.28 ± 1.34	0.108	-0.80 ± 1.37	0.242	-0.42 ± 1.21	0.079	-0.46 ± 1.42	0.221	-0.76 ± 1.04	0.173	6.44 ± 0.59	0.010	46	17.4%	0.066
Yes (*n* = 6)	-1.21 ± 1.05		-1.51 ± 1.49		-1.36 ± 1.16		-1.20 ± 0.97		-1.37 ± 0.68		7.12 ± 0.54		6	50.0%	
Current smoking															
No (*n* = 37)	-0.60 ± 1.40	0.085	-0.90 ± 1.40	0.894	-0.61 ± 1.23	0.509	-0.69 ± 1.35	0.233	-0.91 ± 0.95	0.397	6.59 ± 0.63	0.201	37	27.0%	0.103
Yes (*n* = 15)	0.11 ± 1.38		-0.84 ± 1.40		-0.35 ± 1.25		-0.18 ± 1.46		-0.64 ± 1.19		6.34 ± 0.57		15	6.7%	

### RDS and mechanical ventilation

RDS, diagnosed in 28.8% subjects, was associated with a higher incidence of pulmonary dysfunction in young survivors, as demonstrated by LCI ≥ 6.99 (40%), compared to subjects without this complication (13.5%, *p* = 0.034).

Among subjects treated with surfactant (21.2%) ventilation inhomogeneity (LCI ≥ 6.99) was presented in 45.5% compared to 14.6% not receiving such treatment (*p* = 0.026). Survivors who received surfactant had, on average, a higher LCI 2.5% norm than the others (*p* = 0.010, Table 3). In these participants, there was no significant difference between the groups in terms of airway obstruction evaluated by FEV_1_ < 80%.

Patients mechanically ventilated ≥ 7 days more often had LCI ≥ 6.99 (50.0%) compared to unventilated or ventilated < 7 days (12.5%; *p* = 0.005). Survivors mechanically ventilated longer had on average higher LCI, RV z-score, RV/TLC z-score, and functional residual capacity (FRC) z-score than the others (*p* = 0.025, *p* = 0.053, *p* = 0.020, and *p* = 0.062 respectively, Tables 3 and 4).

**Table 4:** Perinatal and adulthood risk factors and are standardised plethysmographic, IOS, and FeNO tests’ results in prematurely born young adults

	TLC z -score (*n* = 50)		RV z -score (*n* = 51)		RV/TLC z -score (*n* = 50)		FRC z -score (*n* = 51)		Fres (IOS) (*n* = 52)		FeNO (*n* = 50)	
	mean ± SD	p-value	mean ± SD	p-value	mean ± SD	p-value	mean ± SD	p-value	mean ± SD	p-value	mean ± SD	p-value
Prenatal steroids												
No (*n* = 14)	0.45 ± 0.99	0.334	1.04 ± 1.23	0.472	1.03 ± 1.58	0.107	0.64 ± 0.92	0.565	13.49 ± 6.02	0.092	26.07 ± 22.74	0.046
Yes (*n* = 38)	0.75 ± 0.94		0.80 ± 1.02		0.43 ± 0.96		0.46 ± 0.97		11.26 ± 3.24		16.72 ± 9.73	
Congenital pneumonia												
No (*n* = 34)	0.73 ± 1.00	0.536	0.77 ± 1.12	0.418	0.41 ± 1.01	0.134	0.34 ± 1.03	0.079	12.28 ± 4.84	0.328	21.19 ± 17.19	0.247
Yes (*n* = 18)	0.55 ± 0.87		1.03 ± 0.99		0.93 ± 1.38		0.83 ± 0.70		11.06 ± 2.67		16.06 ± 9.26	
Mechanical ventilation												
< 7 days (*n* = 40)	0.67 ± 1.02	0.984	0.71 ± 1.08	0.053	0.39 ± 1.05	0.020	0.38 ± 1.01	0.062	12.10 ± 4.57	0.454	20.69 ± 15.96	0.231
≥ 7 days (*n* = 12)	0.66 ± 0.68		1.41 ± 0.89		1.34 ± 1.35		0.98 ± 0.43		11.05 ± 2.80		14.55 ± 9.60	
Surfactant												
No (*n* = 41)	0.69 ± 1.02	0.765	0.83 ± 1.10	0.688	0.53 ± 1.13	0.546	0.45 ± 0.99	0.369	11.75 ± 4.56	0.730	19.30 ± 15.69	0.970
Yes (*n* = 11)	0.58 ± 0.62		0.99 ± 1.00		0.79 ± 1.35		0.76 ± 0.75		12.26 ± 2.75		19.50 ± 12.09	
RDS												
No (*n* = 37)	0.66 ± 1.06	0.881	0.91 ± 1.11	0.576	0.63 ± 1.12	0.631	0.50 ± 1.01	0.840	11.89 ± 4.73	0.943	19.97 ± 16.31	0.636
Yes (*n* = 15)	0.70 ± 0.58		0.72 ± 0.97		0.44 ± 1.33		0.56 ± 0.79		11.79 ± 2.73		17.71 ± 10.95	
Sepsis												
No (*n* = 46)	0.67 ± 0.99	0.964	0.87 ± 1.10	0.935	0.57 ± 1.15	0.780	0.50 ± 0.96	0.787	11.75 ± 4.37	0.602	19.18 ± 15.23	0.842
Yes (*n* = 6)	0.65 ± 0.74		0.83 ± 0.87		0.71 ± 1.42		0.61 ± 0.91		12.72 ± 3.02		20.50 ± 13.72	
Postnatal steroids												
No (*n* = 47)	0.69 ± 0.99	0.512	0.82 ± 1.05	0.322	0.52 ± 1.11	0.156	0.46 ± 0.96	0.167	11.91 ± 4.37	0.842	19.89 ± 15.26	0.646
Yes (*n* = 4)	0.36 ± 0.47		1.38 ± 1.49		1.39 ± 1.82		1.16 ± 0.61		11.46 ± 3.22		16.25 ± 12.50	
Hospitalisations due to respiratory infections up to 2 years of age												
No (*n* = 40)	0.71 ± 0.97	0.614	0.87 ± 1.12	0.971	0.55 ± 1.17	0.700	0.54 ± 1.01	0.714	11.43 ± 4.42	0.182	17.76 ± 16.31	0.725
Yes (*n* = 12)	0.55 ± 0.95		0.85 ± 0.95		0.70 ± 1.19		0.42 ± 0.78		13.30 ± 3.26		18.00 ± 9.74	
Current asthma												
No (*n* = 46)	0.67 ± 0.97	0.978	0.89 ± 1.11	0.598	0.59 ± 1.14	0.854	0.54 ± 0.97	0.624	11.32 ± 3.02	0.009	18.23 ± 14.08	0.156
Yes (*n* = 6)	0.66 ± 0.94		0.64 ± 0.75		0.50 ± 1.49		0.33 ± 0.77		15.99 ± 8.75		27.50 ± 14.43	
Current smoking												
No (*n* = 37)	0.66 ± 0.96	0.954	0.97 ± 1.07	0.280	0.71 ± 1.25	0.243	0.55 ± 0.88	0.624	12.17 ± 4.72	0.411	16.80 ± 10.52	0.065
Yes (*n* = 15)	0.68 ± 0.98		0.61 ± 1.07		0.29 ± 0.91		0.41 ± 1.11		11.10 ± 2.61		25.27 ± 21.39	

Almost half of the subjects with RDS requiring mechanical ventilation ≥ 7 days had airway obstruction (FEV_1_ / FVC z-score≤-1.646), 66.7% requiring ventilation < 7 days, and 25.9% not ventilated without RDS (p = NS).

Most of the patients (60.0%) with RDS mechanically ventilated for ≥ 7 days and 18.5% of the subjects without RDS and mechanical ventilation had ventilation inhomogeneity (LCI ≥ 6.99). Such disturbances did not occur in patients mechanically ventilated < 7 days (p = NS).

### Congenital pneumonia and sepsis

Survivors with congenital pneumonia more often presented FEV_1_ < 80% pred. (17.6%), FEV_1_/FVC z-score≤-1.646 (35.3%), and LCI ≥ 6.99 (33.3%) than those without such complication: 15.2%; 24.2% and 14.7%, respectively. There was no significant differences between the groups. FEV_1_/FVC z-score≤-1.646 and LCI ≥ 6.99 was observed in half and one third of the patients diagnosed with sepsis in the neonatal period, respectively.

### Hospitalisations due to respiratory tract infections up to 2 years of age

Up to 2 years, hospitalisation due to respiratory tract infections was required by 23.1% of survivors. Hospitalized persons more often presented FEV_1_ < 80% pred (33.3%) than those not hospitalised. It was found a statistically significant association between FEV_1_/FVC z-score ≤-1.646, and hospitalisations due to respiratory tract infections up to 2 years of age (*p* = 0.007). Patients who required such hospitalizations had on average lower FEV_1_/FVC z-score, MEF 75z-score, MEF 50 z-score, and MEF 25 z-score (*p* = 0.013, *p* = 0.006, *p* = 0.006, *p* = 0.010 respectively, Table 3).

Subjects treated with surfactant for RDS required hospitalisation more often (36.4%) compared to those with RDS not receiving surfactant (25.0%) and those without RDS not treated with surfactant (18.9%; p = NS).

Patients requiring mechanical ventilation ≥ 7 days and neonatal surfactant administration were more frequently hospitalised up to 2 years of age for respiratory infections than patients not needing such treatment (33.3% vs. 0.0%; p = NS).

### Asthma, current smoking, and respiratory dysfunction in early adulthood

Adults diagnosed with asthma had more often airway obstruction (FEV_1_/FVC z-score≤-1.646) (66.7% vs. 22.7%, *p* = 0.025), LCI ≥ 6.99 (50% vs. 17.4%, *p* = 0.066), and FeNO > 25 ppb (66.7% vs. 18.2%, *p* = 0.024). Patients who smoked did not have worse results of the lung function tests than non-smokers in terms of the above parameters.

### Lung ultrasound

Single B lines were found in 95% of patients. Only 2 patients had an increased number of these lines (more than 2 in the field of view). In 18.9% of patients, single subpleural consolidations were visible, which did not exceed 6 mm (in the maximum dimension). In 57% of respondents, an irregular or taut outline of the pleural line was found. There were no statistically significant correlations between ultrasound abnormalities (B lines, subpleural consolidations, irregular pleura line, pleural fluid) and GA, FEV_1_ < 80%pred., FEV_1_/FVC ≤-1,646 z-score, LCI ≥ 6.99.

### Quality of life and mental health evaluation

#### QoL, depression and anxiety symptoms

Young adults born prematurely rated their global QoL (Q1) and general health satisfaction (Q2) at a good level (mean 3.98 ± 0.73; mean 3.58 ± 1.01, respectively). Respondents who were born at GA ≤ 28 weeks significantly less frequently described their general QoL as ‘good’ and ‘very good’ in comparison with those born at GA 29–34 (50% vs 84.2%, p = 0.022). Lung tests revealed that participants with LCI ≤ 6.98 more frequently rated their global QoL as ‘good’ and ‘very good’ than those with LCI ≥ 6.99; (83.8% vs. 54.5%, *p* = 0.043). There were no significant associations between the lung function of people born prematurely and their subjective evaluation of QoL in all assessed domains (Table 5).

The PHQ-9 was used to examine the prevalence of depression symptoms, with the mean score of 6.11 ± 5.02. Approximately 10% (10.6%) of the group reported depression symptoms above the cut-off point. To check the severity of anxiety symptoms, the GAD-7 was applied, with a mean score of 5.90 ± 4.78. Anxiety symptoms above the cut-off point were described by 20.8% of respondents. There were no statistically significant differences in the prevalence of depression and anxiety symptoms between subjects born at GA 24–28 and at GA 29–34. Pulmonary function test results showed no association with the severity of depression and anxiety symptoms.

## Discussion

In this prospective cohort study of preterm-born adult survivors at the age of 21 to 24 years, we found that compared with the group born at 29–34 weeks, subjects born at 24–28 weeks of gestation showed a higher incidence of airway obstruction, and higher ventilation inhomogeneity.

As young adults, subjects born prematurely at 24–28 weeks had almost twice more often FEV_1_ less than 80% predicted, compared with those born at 29–34 weeks of GA (25.0% vs. 13.2%). The decreased in FEV_1_ is well supported by a systematic review of 21 publications [30]. The pulmonary outcomes are generally worse among children aged 4–6 years old and also adults who had BPD during the neonatal period [31–33]. In our study, due to the small number of subjects with BPD (*n* = 4), analysis regarding this topic was not performed.

Airway obstruction (FEV_1_/FVC≤-1.646 z-score) was observed in 28.0% of subjects, but only nearly one-half of them (11.5%) are chronically treated for asthma. These data correspond to the results of the multicentre, epidemiological research Epidemiology of Allergic Disorders in Poland (ECAP) in which estimated prevalence of asthma was found in 11% of adults [34].

In our study, airway obstruction was observed in 50% of subjects born at 24–28 weeks and 21.1% in those born at 29–34 weeks of GA. It is in agreement with findings of Harju et al. who noted that children born moderately preterm (≤ 32 weeks gestation) had a significantly increased risk for developing asthma compared to their term-born counterparts [35]. This may indicate that in the group of individuals born prematurely, asthma is underrecognized.

Epidemiological studies show that asthma in preterm-born subjects is likely unrelated to eosinophilic airway inflammation, and a recent systematic review reported that FeNO was low [36]. In our research, we found that elevated FeNO levels (> 25 ppb) were present in 24% of patients and were not associated with airway obstruction. However, they were significantly associated with the diagnosis of asthma (66.7% vs. 18.2%, *p* = 0.024).

The systematic review and meta-analysis indicate that infants born moderate-late preterm (32–37 weeks) had poorer expiratory airflows compared to term-born infants, as per established norms [37]. Preterm-born participants have lower z-scores for FEV_1_, FVC, FEV_1_/FVC, and FEF25-75% than controls. Moreover, preterm-born participants exhibit lower z-scores for FEV_1_, FVC, FEV_1_/FVC, and FEF25-75% compared to the control group. In our study, we also observed lower z-scores for FEV_1_/FVC, in the more premature group, however at the border of significance.

In addition to airway obstruction, gas trapping is seen in preterm-born adults, particularly in those with BPD during the neonatal period [38, 39].

Data regarding the forced oscillation technique shows that preterm children have higher resistance and lower reactance [40]. In our study we haven’t found association between IOS parameters and gestational age. However, the increased value of the Fres was significantly correlated with obstruction in spirometry (FEV_1_ / FVC z–score) (*p* = 0.004). It’s essential to keep in mind that not everyone is suitable for spirometric tests, whereas IOS is comparatively less technically demanding. The correlations we’ve identified suggest that, in specific cases (for example, with children or adults who may have difficulty performing the spirometric test or are uncooperative), spirometry may be replaceable, allowing for the early detection of indications of bronchial obstruction tendencies using IOS. Nevertheless, additional research is required to further investigate this potential.

Alveolar development in the human takes place between 36 weeks of gestation and continues until 18 months after birth [41]. That is why lung injury caused by prematurity, mechanical ventilation, infections, and other factors during this critical period of lung development results in abnormal alveolarisation. This results in disruption of distal lung growth and abnormal microvascular development, which may cause abnormal lung function in adulthood.

Studies evaluating ventilation inhomogeneity and small airway disease in survivors found that LCI was significantly higher in BPD subjects than in non-BPD born preterm and term controls [42–44]. We present that LCI was significantly higher in adults born at 24–28 weeks than those born at 29–34 weeks of gestation (*p* = 0.013). Ventilation inhomogeneity (LCI ≥ 6,99) was detected more often in the more premature group (50% vs. 12,5%). Significantly higher LCI was observed not only in subjects with abnormal FEV_1_ (< 80%pred) but also in 14.3% of those with FEV_1_ ≥ 80%pred. (*p* = 0.021). Caskey et al. reported that 30% of adults with BPD and normal FEV_1_ had abnormal LCI [42]. Furthermore, in our study, this parameter is significantly correlated with GA, and gas trapping (RV/TLC percent and z-score). This indicates that LCI may be more sensitive marker than FEV_1_ to detect early peripheral airway disease in the preterm-born adults’ cohort.

In our study, we observed that prenatal steroids reduce the risk of lung dysfunction in adulthood. Participants who received prenatal steroid treatment had a lower occurrence ofventilation inhomogeneity (LCI ≥ 6.99). However, based on a prospective, multicentre study, prenatal steroids did not impact the incidence of BPD. Nevertheless, they affect its severity, resulting in most patients having mild BPD, and long-term improvements [45].

The widespread administration of prenatal steroids, along with improved quality of ventilation care during the neonatal period, has played a significant role in altering the outcome profiles for infants born in different time periods [30]. Pulmonary function outcomes of a cohort born in 1999–2000 were considerably superior to those born in 1991–1992 [46].

On the other hand, only 4 participants in our study received postnatal steroids. With such a small number of subjects, we can only assume that postnatally steroid-requiring patients tend to have abnormal FEV_1_ (< 80% pred.), obstruction (FEV_1_ / FVC ≤-1.646 z-score), and impaired ventilation homogeneity (LCI ≥ 6.99), but this requires confirmation on a larger group. These patients were in severe clinical condition and also required prenatal steroids.

Likewise, individuals who required surfactant supplementation more frequently exhibited elevated LCI (≥ 6.99), which could be attributed to the presence of emphysema and atelectasis areas during the neonatal period. These factors may subsequently impact ventilation inhomogeneity in the future.

The long-term effectiveness of non-invasive nCPAP, hasn’t been confirmed yet and according to the best current evidence only the volume target ventilation (VTV) has been proved to reduce the risk of BPD or neonatal death in comparison to other methods of ventilation [47]. However, early use of non-invasive support and optimal nutrition plays today a crucial role in BPD prevention [48]. By enhancing the quality of ventilation care during the neonatal period, there is potential for better prevention of ventilation-related disorders (such as emphysema and atelectasis) in adulthood [49, 50].

This is supported by our observation that patients requiring mechanical ventilation for 7 days or more are more likely to have an elevated LCI (*p* = 0.005) These findings further indicate that a longer duration of mechanical ventilation is associated with a higher risk of complications such as volutrauma and barotrauma during the neonatal period, as well as with impaired lung function in adulthood. Conversely, mechanical ventilation for less than 7 days is associated with a lower risk of complications, and late-onset pulmonary dysfunction in adulthood.

Our findings suggest that both RDS and mechanical ventilation for 7 days or more are considered risk factors for obstruction and impaired ventilation homogeneity in adulthood. Therefore, it is important to consider these complications and monitor lung function in the subsequent years of life.

Additionally, we believe that congenital pneumonia and sepsis are factors that worsen the prognosis and contribute to the deterioration of functional parameters of the respiratory system in adulthood, including a tendency towards airway obstruction and ventilation inhomogeneity. Furthermore, congenital pneumonia is associated with reduced FEV_1_ in adulthood. During inflammation and infection, cytokines are released, and they destroy surfactant, thereby increasing the surface tension in the lung alveoli, which promotes their damage.

Preventing infections and minimising hospitalisations during the first two years of life is crucial, as they are associated with an increased risk of airway obstruction in adulthood (*p* = 0.007). Currently, palivizumab is recommended for preterm infants to protect against severe respiratory syncytial virus (RSV) infections and their associated complications [51].

In our study, we found that smokers did not exhibit worse results in PFTs compared to non-smokers. However, it is essential to note that this observation should not be interpreted as a cause-and-effect relationship. It is likely that individuals in poorer clinical condition with pre-existing risk factors may have chosen not to initiate smoking.

It is worth emphasising that despite being born prematurely, the average results of pulmonary function tests in premature-born survivors do not significantly differ from those of the general adult population at the age of 20, as indicated by the z-score values.

Lung ultrasonography, which is increasingly available and utilised in clinical practice, shows promise in diagnosing and predicting the severity of BPD [52]. However, investigation of potential applications of lung ultrasonography for detecting disorders associated with prematurity in young adults in our study, did not reveal any statistically significant associations between ultrasound abnormalities (such as B lines, subpleural consolidations, uneven outline of the pleura, and pleural fluid) and GA or parameters like LCI and FEV1/FVC. Further research is necessary to evaluate alternative imaging techniques, such as low-dose chest CT or MRI, for lung monitoring in adults born prematurely.

Young adults born prematurely rated their global quality of life and health satisfaction well, with a mean rating of ‘good’. Current reports do not provide clear conclusions regarding the comparison of the QoL of adults born prematurely in comparison with those born at term. A systematic review published in 2020 found that out of 18 studies, 11 showed no differences between premature and term-born subjects in terms of QoL. Four studies reported lower QoL in preterm adults, and three studies had inconclusive findings [53]. An interesting finding related to the pulmonary functioning of adults born prematurely was observed in relation to the association between ventilation inhomogeneity and subjective assessment of global QoL. Participants with LCI value of less than 6.98 were significantly more likely to describe their QoL as ‘good’ or ‘very good’ compared to those with an LCI value of 6.99 or higher. No similar association was found with other lung function test results. This leads us to conclude that LCI is the highly sensitive method for assessing lung function, capturing minor changes in respiratory function that are related to the subjective assessment of QoL. To the authors’ knowledge, this association between LCI and subjective QoL has not been previously reported.

The prevalence of depressive and anxiety symptoms was measured by short screening tests. The study revealed that 10.6% of the respondents exhibited elevated symptoms of depression, while 20.8% had severe symptoms of anxiety. It is important to note that the study was conducted during the COVID-19 pandemic, which could have had an impact on the mental health of the participants. Therefore, it is crucial to compare these results with those of a healthy population who were also evaluated during the pandemic to gain a better understanding of the findings. The study conducted by Gambin et al. in Poland during a similar period (May 2020) utilized the same screening tools, PHQ-9 and GAD-7. Their findings revealed that among healthy individuals, 25.3% of men and 29.7% of women reported clinically significant severity of anxiety symptoms. Furthermore, 24.6% of men and 25.3% of women reported clinically significant severity of depressive symptoms [54]. The lower number of reported symptoms of depression and anxiety in our study group may be attributed to the enhanced adaptive, coping, and resilience skills of adults born prematurely [53].

Preterm birth should be recognised as a chronic condition, as some individuals who survive are at risk of developing chronic obstructive airway disease later in life. Therefore, gaining a comprehensive history of GA, course, and complications during the neonatal period should be a routine part of care for adult patients. Preterm birth should no longer be seen solely as a challenge for pediatricians, but also for adult pulmonologists who now care for an increasing population of adult survivors.

With the advancements in medical and technological support for respiratory care, the proportion of premature infants requiring intubation and mechanical ventilation has significantly decreased compared to previous decades [48]. It would be valuable to replicate the study in premature infants born in the present era to assess current outcomes and implications, considering the progress in medical care and respiratory support.

Strengths and Limitations.

A strength of this study is its ability to track and examine a carefully characterised group of preterm infants into adulthood. Individuals were personally examined and followed from birth by a team led by one of the authors. The publication incorporates unique clinical data from 20 years ago, as well as modern respiratory system diagnostic methods like MBNW and psychological tests. The combination of new technology with assessments of the quality of life adds originality and comprehensiveness to the approach. The limitation of the study was the small sample size, which was not enough powerful to reject some bivariate statistical hypotheses and precluded an in-depth analysis of multiple variables at once. Regarding the selection bias, the subjects included in the study exhibited a high degree of similarity both among themselves and in comparison to the overall population of newborns from which they were drawn. There were no discernible systematic differences between the study groups. When comparing the group of newborns born between 24 and 32 weeks of gestation in 1999–2000 who were discharged from the hospital (*n* = 244) with the study group (*n* = 52), no evidence of sample selection was found. Upon comparing various factors, such as gestational age, average pregnancy duration, gender, whether the pregnancies were single or multiple, maternal age, education, per capita income, and place of residence, no statistically significant differences were observed between the group of all premature infants discharged from the hospital and the current study group after 20 years. Cerebral palsy was diagnosed in 14 premature infants, out of which two volunteered for participation in our study. However, due to the lack of cooperation, respiratory function tests could not be conducted. Since patient cooperation was one of the inclusion criteria, it is not possible to determine the impact of cerebral palsy on the results of the pulmonary function tests.

## Conclusions

In the era of enhanced care for preterm-born infants, there is a growing interest in monitoring the long-term health of adult survivors who are at risk of developing lung function abnormalities. Preterm adults born at GA of 28 weeks or less, with a history of RDS, surfactant administration, postnatal steroid use, pneumonia, and hospitalisation due to respiratory infections in their first two years of life, face an increased risk of impaired lung function compared to those born between 29 and 34 weeks of gestation.

Consequently, it is vital to routinely gather comprehensive birth history to facilitate early diagnosis and prompt intervention in adulthood.

Regular follow-up evaluations should include assessments of lung function to evaluate airway obstruction and ventilation inhomogeneity. In particular, the utilisation of MBNW testing may offer new insights into long-term pulmonary impairment in adults born prematurely. LCI have shown promise as sensitive indicator of ventilation inhomogeneity, which correlates with GA and neonatal risk factors. It is crucial to establish standards for early prevention, detection, and monitoring of disorders associated with prematurity in adult survivors. Furthermore, additional longitudinal studies are necessary to investigate outcomes beyond the second decade of life. These studies should aim to identify risk factors and develop optimal treatment strategies for the long-term consequences of prematurity.

**Table 5:** Quality of life, depression and anxiety symptoms by gestational age and pulmonary function tests’ results in prematurely born young adults

	WHOQoL (*n* = 48)												PHQ-9 Depression symptoms ≥ 12 (*n* = 47)		GAD-7 Anxiety symptoms ≥10 (*n* = 48)	
	Domains								Global QoL (Q1)		Health Satisfaction (Q2)					
	Physical health		Psychological		Social relationships		Environment									
	mean ± SD	p-value	mean ± SD	p-value	mean ± SD	p-value	mean ± SD	p-value	mean ± SD	p-value	mean ± SD	p-value	%	p-value	%	p-value
**Gestational age**																
24–28 (*n* = 10)	16.30 ± 2.63	0.323	15.00 ± 2.54	0.918	15.60 ±2.91	0.788	16.60 ±1.71	0.719	3.80 ± 0.92	0.334	3.30 ± 1.34	0.403	20.0%	0.279	20.0%	0.942
29–34 (*n* = 38)	15.50 ± 2.72		14.76 ± 3.12		15.37 ±3.57		15.97 ±2.35		4.03 ± 0.68		3.66 ± 0.91		8.1%		21.1%	
**Pulmonary function**																
FEV1%																
$\ge$80% (*n* = 39)	15.46 ± 2.92	0.398	14.61 ± 3.19	0.926	15.10 ± 3.67	0.363	15.90 ± 2.35	0.182	3.95 ± 0.76	0.906	3.51 ± 1.05	0.770	13.2%	0.309	25.6%	0.130
< 80% (*n* = 7)	16.71 ± 0.95		15.00 ±1.15		16.71 ± 1.70		17.15 ± 0.69		4.00 ± 0.58		3.71 ± 0.76		0%		0%	
FEV_1_/FVC z-score																
>-1.645 (*n* = 33)	15.47 ± 2.96	0.888	14.76 ± 3.27	0.324	15.20 ± 3.71	0.940	15.82 ± 2.31	0.204	4.00 ± 0.74	0.423	3.50 ± 1.05	0.740	12.1%	0.721	23.5%	0.620
≤-1.646 (*n* = 12)	16.17 ± 2.04		14.41 ± 1.93		15.75 ± 2.83		16.83 ± 1.80		3.83 ± 0.72		3.67 ± 0.89		8.3%		16.7%	
LCI 2.5%norm.																
< 6.98 (*n* = 37)	15.57 ± 2.91	0.910	14.84 ± 3.26	0.414	15.86 ± 3.39	0.067	16.05 ± 2.36	0.941	4.03 ± 0.69	0.350	3.57 ± 0.99	0.855	11.1%	0.849	24.3%	0.275
≥ 6.99 (*n* = 10)	16.00 ± 1.84		14.73 ± 1.90		13.91 ± 3.21		16.27 ± 1.85		3.82 ± 0.88		3.64 ± 1.12		9.1%		9.1%	
FeNO																
≤ 25 ppb (*n* = 36)	15.44 ± 2.86	0.286	14.50 ± 3.02	0.264	15.31 ± 3.44	0.713	15.94 ± 2.32	0.656	3.92 ± 0.73	0.512	3.44 ± 1.00	0.180	14.3%	0.317	25.0%	0.413
> 25 ppb (*n* = 11)	16.27 ± 2.15		15.45 ± 2.73		15.64 ± 3.64		16.36 ± 1.91		4.09 ± 0.70		3.91 ± 0.94		0.0%		9.1%	

## Data Availability

The datasets used and analysed during the.current study available from the corresponding author on reasonable request.
